# A magneto-thermoelectric with a high figure of merit in topological insulator Bi_88_Sb_12_

**DOI:** 10.1038/s41563-024-02059-9

**Published:** 2025-01-03

**Authors:** Yu Pan, Bin He, Xiaolong Feng, Fan Li, Dong Chen, Ulrich Burkhardt, Claudia Felser

**Affiliations:** 1 Max Planck Institute for Chemical Physics of Solids, Dresden, Germany; 2 College of Materials Science and Engineering and Center of Quantum Materials & Devices, Chongqing University, Chongqing, People’s Republic of China; 3 NISE Department, Max Planck Institute of Microstructure Physics, Halle, Germany; 4 College of Physics and Center of Quantum Materials & Devices, Chongqing University, Chongqing, People’s Republic of China

**Keywords:** Thermoelectrics, Topological insulators

## Abstract

High thermoelectric performance is generally achieved by synergistically optimizing two or even three of the contradictorily coupled thermoelectric parameters. Here we demonstrate magneto-thermoelectric correlation as a strategy to achieve simultaneous gain in an enhanced Seebeck coefficient and reduced thermal conductivity in topological materials. We report a large magneto-Seebeck effect and high magneto-thermoelectric figure of merit of 1.7 ± 0.2 at 180 K and 0.7 T in a single-crystalline Bi_88_Sb_12_ topological insulator. This result fills a gap of a high performance below 300 K and is promising for low-temperature thermoelectric applications. The large magneto-Seebeck response is attributed to the ultrahigh mobility and the Dirac band dispersion. The application of a low magnetic field to achieve a high thermoelectric performance can be extended to topological materials with similar features that are rapidly emerging because it synergistically optimizes the thermoelectric parameters.

## Abstract (web summary)

Applying a magnetic field to the topological material Bi_88_Sb_12_ enhances the Seebeck coefficient, resulting in a high thermoelectric figure of merit *zT* of 1.7 at 180 K and 0.7 T.

## Main

Topological materials with unique band structures have attracted much attention in condensed-matter physics, thermoelectrics, spintronics and so on^1–4^. Thermoelectric technology, owing to the ability to directly convert heat into electricity or vice versa, is of great significance for solving the global energy crisis and implementing solid-state cooling^5^. The thermoelectric energy conversion efficiency depends on the thermoelectric figure of merit *zT*, defined as *zT* = (*α*^2^/*ρκ*)*T*, where *α* is the Seebeck coefficient, *ρ* is the resistivity, *κ* is the thermal conductivity and *T* is the absolute temperature^5,6^. The beneficial properties of topological materials, such as linear band dispersion, small Fermi surfaces, ultrahigh mobility, robust topologic states and heavy elements, provide a great opportunity to explore high *zT* values in topological materials^7–12^. On one hand, a handful of examples have shown that the topological states induce a large Berry curvature, which can lead to strong transverse anomalous Nernst signals^7–10^. On the other hand, many topological insulators share the same features, such as a narrow bandgap and small effective mass, as thermoelectric semiconductors, thus presenting a commendable thermoelectric performance based on the Seebeck effect.

Searching for a pathway to enhance the *zT* of topological materials by synergistically tuning the coupled thermoelectric parameters has attracted increasing attention. One effective way is to enhance the Seebeck coefficient since *zT* is proportional to the square of the Seebeck coefficient, particularly when the topological materials already show low resistivity and low thermal conductivity due to a sharp band dispersion and heavy elements, respectively. Enhancing the Seebeck coefficient is usually realized by band engineering^13^, by an ‘energy filtering’ effect^14,15^ or possibly by applying a magnetic field as found in various topological materials^16–18^. Notably, magneto-thermoelectrics are attracting wide attention with the surge in topological materials. This is because, as theoretically proposed, the Seebeck coefficient is enhanced by a weak magnetic field in Dirac and Weyl materials with a linear band dispersion; these materials even show a non-saturating magnetic field dependence when reaching the extreme quantum limit^11,12^. In fact, since 88% of inorganic compounds have been found to have topological bands, a great opportunity exists to realize a high magneto-*zT* in topological materials^19^.

Bismuth-rich Bi_1–*x*_Sb_*x*_ alloys, as renowned topological insulators with Dirac band dispersion and tiny Fermi surfaces^20,21^, are ideal candidates to realize a strong magneto-thermoelectric response. Because of their rich physical phenomena, exploring the properties of Bi_1–*x*_Sb_*x*_ alloys has held the interest of researchers in condensed-matter physics and materials science. Investigations of the thermoelectric performance of Bi_1–*x*_Sb_*x*_ alloys date back to the 20th century^22–24^. The historical record *zT* value is 1.28 at ~225 K under a magnetic field of 1.7 T; however, the field and temperature dependence of the thermoelectric properties as well as the reasons for the large magneto-thermoelectric effect were not described in detail^23^. Rare reports of Bi_1–*x*_Sb_*x*_ have shown a high *zT* value (above 1.2) below 1 T, and no other materials have shown a *zT* value above 1 below 300 K. Understanding the mechanisms of the large magneto-thermoelectric effect and finding a way to realize a high *zT* of Bi_1–*x*_Sb_*x*_, particularly at a low magnetic field such as that produced by a permanent magnet, is important for the thermoelectric cooling market.

Herein we report that a small magnetic field, below 1 T, is able to greatly enhance the *zT* by nearly two to three times in topological insulator Bi_88_Sb_12_, leading to a maximum *zT* of ~1.7 ± 0.2 at around 180 K and 0.7 T. The large enhancement in magneto-*zT* originates from the enhanced magneto-Seebeck coefficient and the decreased magneto-thermal conductivity, which overcomes the magneto-resistivity. Theoretical analysis shows that the magneto-Seebeck coefficient is closely related to the energy dependence of the cyclotron frequency and the change of Fermi level due to Zeeman splitting. The result can be extended to other topological materials with sharp band dispersions, because it leads to a large magneto-Seebeck effect and therefore enhances the *zT* value.

## Linear band dispersion enhances magneto-*zT* at low fields

**Fig. 1:**
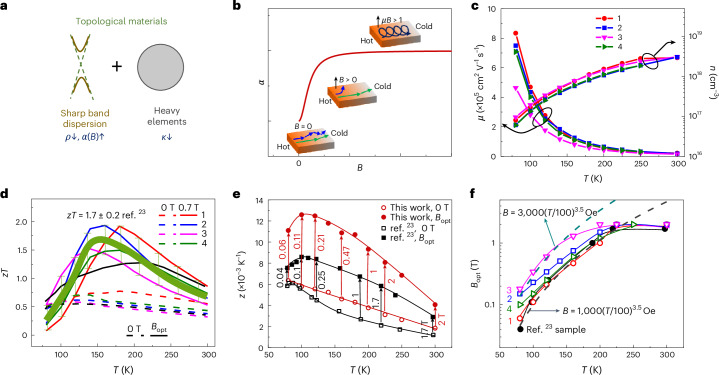
Large magneto-*zT* in Bi_88_Sb_12_ topological insulator. **a**, Two important features of topological materials are their sharp band dispersion and heavy elements. **b**, Schematic illustration of the Seebeck coefficient as a function of magnetic field. Insets show that the magneto-Seebeck coefficient saturates until the electrons achieve a cyclotron motion. **c**,**d**, Temperature dependence of charge carrier mobility *μ* and concentration *n* (**c**) and of *zT* values (**d**) measured in four different samples. The light green line in **d** shows the mean *zT* values of the four samples, showing an average peak *zT* of 1.7 with an error within 20% for the four samples. The error bars present the *zT* variation between the four samples. **e**, Temperature dependence of the thermoelectric *z* value of sample no. 1 compared to the reported record value^23^. The vertical arrows present the magnetic field for achieving the *z* enhancements. **f**, Temperature dependence of the optimal magnetic field required for maximum *z* values in four different samples. Source data

We propose magneto-thermoelectric correlation as an effective strategy to tune the thermoelectric parameters of topological materials. As schematically shown in Fig. 1a, topological materials usually have two important features: they have a linear band dispersion and are composed of heavy elements. The sharp band dispersion guarantees high mobility, resulting in low resistivity and a large magneto-Seebeck effect; the heavy elements produce a low thermal conductivity. Given the cost of the magnetic field for practical applications, achieving a large magneto-thermoelectric performance at a relatively low magnetic field that can be achieved using a permanent magnet, is important. This requires the material to show a strong magneto-thermoelectric response, particularly a pronounced magneto-Seebeck effect.

A large magneto-Seebeck effect at a low magnetic field can be understood in two ways. On one hand, the magnetic field shows a preferential influence on charge carriers with different energies^25^, which acts as an ‘energy filtering’ effect. As shown in Fig. 1b, the Seebeck coefficient is enhanced until the electrons are forced into a cyclotron motion when *μB* > 1, where *μ* and *B* are the charge carrier mobility and magnetic field, respectively. Then the magneto-Seebeck coefficient reaches the saturation value *α*_∞_, which can be regarded as a Seebeck coefficient without any scattering, as the mean free path becomes the cyclotron radius. By semiclassical transport theory, the magneto-Seebeck coefficient is generally described by *α* = *α*_∞_*μ*^2^*B*^2^/(1 + *μ*^2^*B*^2^) + *α*_0_/(1 + *μ*^2^*B*^2^), where *α*_0_ is the Seebeck coefficient at zero magnetic field, assuming that resistivity *ρ* is magnetic-field independent^26,27^. In this case, the magneto-*zT* is affected only by magneto-*α*. For topological materials with high *μ*, *ρ* is usually magnetic-field dependent, and the magneto-resistivity is large. The increase of *α*, decrease of electronic thermal conductivity and increase of *ρ* compete in a delicate balance, which can lead to an increase of the power factor and *zT* when a high *μ*, large ratio of *α*_∞_/*α*_0_ and low lattice thermal conductivity exist (Supplementary Fig. 1). On the other hand, the magnetic field affects the Seebeck coefficient since the cyclotron frequency of the charge carriers is energy dependent. For an arbitrary band dispersion *ε*(*p*) ∝ *p*^*γ*^, the Boltzmann equation can lead to a cyclotron frequency $\omega (\varepsilon )={eB}\frac{{\varepsilon }^{{\prime} }\left(p\right)}{p}{|}_{p=p(\varepsilon)}$, and the Seebeck coefficient is enhanced for *γ* < 2, is suppressed for *γ* > 2 and remains constant for *γ* = 2 (ref. ^11^), in which *ε*, *p*, *γ*, *ε*' and *e* are energy, momentum, index number, energy differential and elementary charge, respectively. Therefore, topological materials with their linear band dispersion and heavy elements, which contribute to high mobility, a large magneto-Seebeck effect and a low lattice thermal conductivity, are of great interest to achieve a high magneto-thermoelectric performance.

Four different samples were studied to demonstrate the critical role of mobility for achieving large magneto-*zT* values. As shown in Fig. 1c, the high mobility and low charge carrier concentration indicate the high purity of the crystals used. Moreover, the four samples present almost identical charge carrier concentrations with only small differences, while some variation exists between the mobilities of the four samples. Figure 1d compares the *zT* values of the four samples under 0 T and 0.7 T. Two of the samples achieve a maximum *zT* of ~2, two samples show a lower maximum *zT* of ~1.5 (detailed thermoelectric properties are shown in Supplementary Figs. 2–4) and the average maximum *zT* value is about 1.7, with an ~20% error among the four samples. All the samples show a better *zT* (Fig. 1d) and *z* (Fig. 1e and Supplementary Figs. 5 and 6) compared to previous values^23^, and the optimal field *B*_opt_ to obtain the maximum *z* value (*z*_max_) is found to decrease rapidly with decreasing temperature. Previously, Wolfe and Smith proposed that *B*_opt_ = 1,000(*T*/100)^3.5^ (ref. ^23^). In this work, by investigating more samples, we found that *B*_opt_ = *A*(*T*/100)^3.5^, in which *A* ranges from 1,000 to 3,000. At low temperatures, a small *B*_opt_ is required for *z*_max_ because the magneto-Seebeck coefficient reaches a peak rapidly; otherwise, the magneto-resistivity is too large to decrease *z*. At higher temperatures, since the magneto-resistivity is low, the magneto-Seebeck coefficient plays a dominant role in enhancing *z*, and a larger *B*_opt_ is required for *z*_max_ until the magneto-Seebeck coefficient reaches its peak. Moreover, the variations of *A* indicate that *B*_opt_, and therefore *zT*, has some degree of sample dependence, probably due to the complicated competition between the magneto-Seebeck behavior and the different magneto-resistivity behaviour (Supplementary Fig. 7), both of which are very sensitive to the mobility, band dispersion, Fermi level and so on and worth future study. Most importantly, *B*_opt_ for the maximum *zT* value (*zT*_max_) in this work is largely reduced; the high *zT* of 1.7 ± 0.2 at 180 K and 0.7 T makes Bi_88_Sb_12_ a great candidate for thermoelectric cooling below 300 K.

## Magnetic-field-dependent thermoelectric transport properties

**Fig. 2:**
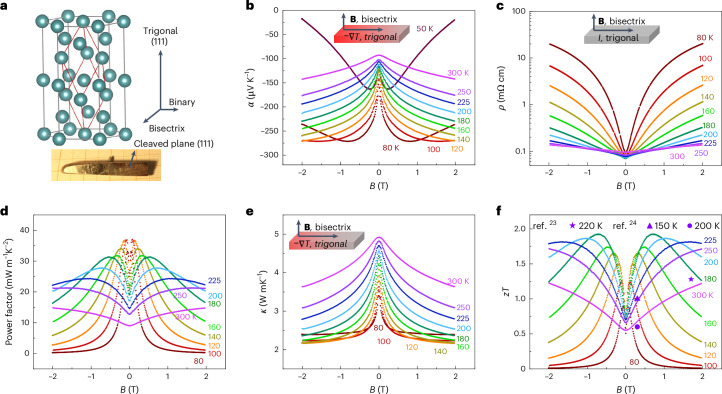
Crystal structure and magneto-thermoelectric properties. **a**, Crystal structure of Bi-rich Bi_1–*x*_Sb_*x*_. The bottom panel shows the optical image of the as-grown Bi_88_Sb_12_ single crystal with a shining cleavage plane in the (111) orientation. **b**–**f**, Seebeck coefficient (**b**), resistivity (**c**), power factor (**d**), total thermal conductivity (**e**) and *zT* (**f**) as a function of magnetic field. Insets in **b**, **c** and **e** illustrate the directions of magnetic field **B** and temperature gradient ∇*T* or current *I*. The power factors and *zT* values show peak values at lower magnetic fields with decreasing temperature. Source data

Bi-rich Bi_1–*x*_Sb_*x*_ alloys crystallized in a rhombohedral structure with a space group of *R*-3*m* (similar to pure Bi) and high-quality Bi_1–*x*_Sb_*x*_ single crystals can be easily cleaved along the (111) plane (Fig. 2a and Supplementary Fig. 8). The Bi/Sb ratio of the crystals was determined to be approximately 88:12 using energy dispersive X-ray spectroscopy and electron backscatter diffraction (Supplementary Figs. 9 and 10). The transport properties of our Bi_88_Sb_12_ samples showed a strong magnetic-field dependence. The Seebeck coefficient was greatly enhanced by an external magnetic field as low as 0.1 T (Fig. 2b) and exhibited a saturation trend at an elevated magnetic field above 80 K. Meanwhile, a large positive field dependence of the resistivity was observed (Fig. 2c). The variation of magneto-resistivity shrinks with increasing temperature due to decreased mobility. Because the large increase in absolute value of Seebeck coefficient surpassed the increase in resistivity, the power factor was greatly enhanced, exhibiting a sharp increment with a peak value below 1 T. The peak value of the power factor shifted towards a higher magnetic field at higher temperatures, as shown in Fig. 2d. In addition, due to the large magneto-resistivity, a reduction in magneto-thermal conductivity was observed (Fig. 2e), and the saturation value can be regarded as the lattice contribution. The enhanced power factor and reduced thermal conductivity promise a large magneto-*zT* value below 1 T. Compared to previously reported values^23,24^, including the historical record (*zT* = 1.28 at ~225 K and 1.7 T; ref. ^23^), the present samples show a maximum magneto-*zT* value of approximately 2 at 180 K and 0.7 T, as shown in Fig. 2f.

## Temperature-dependent thermoelectric transport properties

**Fig. 3:**
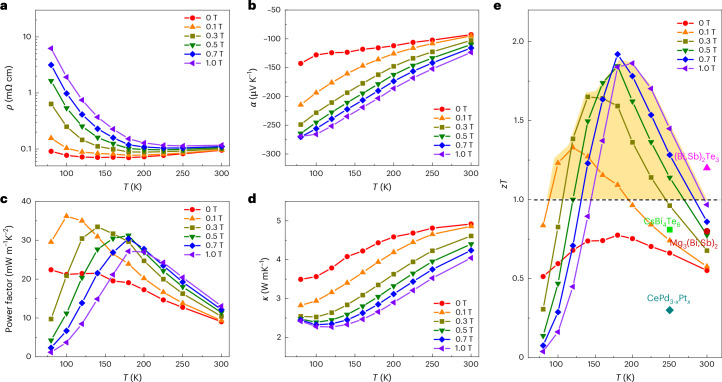
Temperature dependence of thermoelectric transport properties. Thermoelectric transport properties of the Bi_88_Sb_12_ single crystal in the range of 0–1 T. **a**, Resistivity. **b**, Seebeck coefficient. **c**, Power factor. **d**, Total thermal conductivity. **e**, The *zT* values. High *zT* values, above 1, were achieved in a wide temperature range of 100–300 K with a low magnetic field of less than 1 T, as shown by the dashed line and yellow area in **e**. Compared to other thermoelectric materials with high performance near and below 300 K, including (Bi,Sb)_2_Te_3_, CsBi_4_Te_6_, Mg_3_(Bi,Sb)_2_ and CePd_3–*x*_Pt_*x*_, the present study’s Bi_88_Sb_12_ single crystal presents a greatly enhanced *zT*. Source data

Figure 3 shows the temperature dependence of thermoelectric transport properties from 0 to 1 T. Upon cooling, resistivity exhibited a semiconductor transport behaviour and increased with increasing magnetic field (Fig. 3a). The increasing trend above 200 K is attributed to the enhanced phonon scattering. The absolute value of Seebeck coefficient decreases with increasing temperature due to thermal excitation as a result of the small bandgap in Bi_88_Sb_12_ (Fig. 3b). The field-enhanced absolute value of Seebeck coefficient becomes smaller at higher temperatures. Since the maximum absolute value of magneto-Seebeck coefficient and the maximum magneto-resistivity are both at low temperatures, the power factors present a complex behaviour. As shown in Fig. 3c, the power factors are enhanced in the entire temperature range from 80 K to 300 K under a small magnetic field of 0.1 T. Strikingly, the power factor was greatly enhanced from 22 mW m^–1^ K^–2^ at 0 T to 37 mW m^–1^ K^–2^ at 0.1 T near 100 K. The peak power factors shift to higher temperatures with increasing field. At temperatures above 150 K, the power factor under a magnetic field is always higher than that at zero field. The total thermal conductivity increases with temperature due to thermal excitation, as shown in Fig. 3d. Consequently, due to the enhanced power factor and reduced total thermal conductivity, the *zT* values were remarkably enhanced. As shown in Fig. 3e, *zT* attained a maximum value of 0.7 at ~150 K and 0 T, which was enhanced to 1.3 by a low magnetic field of only 0.1 T and further increased to ~1.9 at ~180 K and 0.7 T.

Currently, high *zT* values (above 1) have been reported for numerous compounds at intermediate and high temperatures, and only a few materials exhibit *zT* values near 2 above 600 K (refs. ^28,29^). However, the development of high-performance materials at low temperatures for thermoelectric cooling, particularly near or below 300 K, is unsatisfactory. Here we demonstrate that the Bi_88_Sb_12_ single crystal exhibits a high *zT* value (above 1) in a wide temperature range of 150–300 K; this value is much higher than those of other low-temperature thermoelectric materials^30–33^ and on par with those of intermediate-temperature and high-temperature thermoelectric materials. The high *zT* values (above 1) achieved over the wide temperature range of 150–300 K fill the gap that existed for high-performance thermoelectric materials below 300 K, which can be useful for thermoelectric cooling at low temperatures.

## Band structure analysis and magneto-Seebeck modelling

**Fig. 4:**
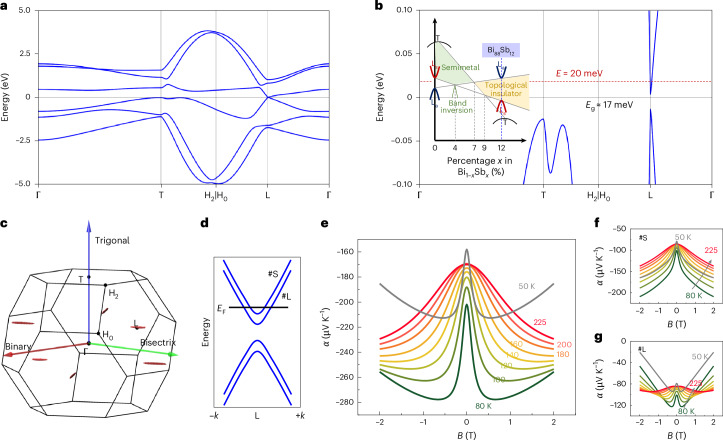
Band structure of Bi_88_Sb_12_. **a**, Band structure of Bi_88_Sb_12_ under zero magnetic field. **b**, An enlarged view of the band structure close to the Fermi level at the T and L points. Inset shows schematic of the composition dependence of the band structures of Bi_1–*x*_Sb_*x*_, with *x* ranging from 0 to 12%. A band gap *E*_g_ of around 17 meV is found in Bi_88_Sb_12_. **c**, Brillouin zone and electron Fermi pockets with a Fermi energy at 20 meV. **d**, Schematic illustration of the band dispersion with Zeeman splitting. A large *g*-factor in Bi_1–*x*_Sb_*x*_ would split the degenerate Fermi surfaces under zero field at L points into two individual pockets, one that is smaller inside (marked #S) and another one that is larger outside (marked #L). The *E*_F_ is reduced for the #S band and increased for the #L band compared to that in the degenerate state. Here *k* is a wave vector. **e**–**g**, Calculated Seebeck coefficient under Zeeman splitting: total Seebeck coefficient (**e**), Seebeck coefficient contributed by #S band (**f**) and Seebeck coefficient contributed by #L band (**g**). The total Seebeck coefficient presents a fallback after reaching the maximum at lower temperatures, in good agreement with the experiment. Source data

Bi_88_Sb_12_ is a gapped system with the band extrema lying at the L point, as shown in Fig. 4a,b. The alloying of Sb changes Bi_1–*x*_Sb_*x*_ (*x* < 0.22) from a semimetal to a topological insulator. As shown in the inset in Fig. 4b, at *x* ≈ 4%, the gap between the L_s_ and L_a_ bands closes, resulting in a fourfold degenerate Dirac point. When *x* ≈ 7%, the overlap between the T and L bands disappears and Bi_1–*x*_Sb_*x*_ alloys transform into inverted-band insulators. On further increasing *x*, Bi_1–*x*_Sb_*x*_ alloys become direct-gap topological insulators, with the T band lying below the L_s_ band^34^. In fact, the origin of various peculiar properties of the system can be attributed to Dirac electrons with a linear band dispersion and extremely high mobility. Figure 4c shows the Fermi pockets in the Brillouin zone at energy *E* = 20 meV, wherein three electron pockets at the L point with strong anisotropy lie along the bisectrix direction, with a small tilt angle (~6° in pure Bi) off the binary–bisectrix plane^35^. The small Fermi surface leads to a low charge carrier concentration. The highly anisotropic Fermi surfaces make Bi_88_Sb_12_ show the highest mobility along the trigonal direction, for which the best magneto-thermoelectric performance is achieved in the trigonal direction.

Under a magnetic field, the large Landé *g*-factor in Bi_1–*x*_Sb_*x*_ splits the degenerate Fermi surfaces under zero field at the L points into two individual pockets, which essentially affects the Seebeck coefficient. Taking a *g*-factor of about 120–240 (ref. ^36^), a Zeeman splitting energy of about 5–10 meV exists at 0.7 T, which is comparable to the thermal broadening energy at 80 K. This indicates that both of the individual pockets, the smaller inside (#S) and the larger outside (#L) pockets, contribute to the conduction, but with different Fermi levels (Fig. 4d). Using a massive Dirac Hamiltonian^37^, the magneto-Seebeck coefficient at different temperatures is calculated^11^:

$$\alpha=\frac{{\uppi }^{2}{k}_{{\mathrm{B}}}^{2}T}{3e}\frac{{E}_{{\mathrm{F}}}}{\left({E}_{{\mathrm{F}}}^{2}-{\Delta }^{2}\right)}\frac{2+{\left(\Delta /{E}_{{\mathrm{F}}}\right)}^{2}+3{\omega }_{{\mathrm{c}}}^{2}{{\rm{\tau }}}^{2}}{1+{\omega }_{{\mathrm{c}}}^{2}{{\rm{\tau }}}^{2}},$$

where the cyclotron frequency ${\omega }_{{\mathrm{c}}}=\frac{{eB}{v}_{{\mathrm{f}}}^{2}}{{E}_{{\mathrm{F}}}}$ and *ν*_f_ is Fermi velocity, *k*_B_, Δ and *τ* are Boltzmann constant, half of the energy gap and relaxation time, respectively. The calculated Seebeck coefficients without and with Zeeman splitting are shown in Supplementary Fig. 11 and Fig. 4e. Without Zeeman splitting (Supplementary Fig. 11), the Seebeck coefficient presents an enhancement and reaches saturation due to the increased cyclotron frequency. With the presence of Zeeman splitting, as shown in Fig. 4e, one can observe a substantial reduction after reaching a maximum at low temperatures, and the peak structure is more pronounced at lower temperatures, which agrees well with experiment (Fig. 2b). The distinct qualitative differences, particularly the peak structure, in the magneto-Seebeck coefficient from those of other Dirac systems like Cd_3_As_2_ demonstrate the essential role of the Zeeman splitting effect in tuning the magneto-Seebeck effect of Bi_1–*x*_Sb_*x*_. The Zeeman splitting effect results in two pockets, and each of them has an individual contribution to the magneto-Seebeck effect, as shown in Fig. 4f,g. As the field increases, the small pocket #S shows a decrease in *E*_F_, together with an increasing cyclotron frequency, indicating a monotonically increasing Seebeck coefficient. By contrast, for the large pocket #L, a continuous increase in *E*_F_ exists. Meanwhile, the cyclotron frequency also increases, leading to a competition curve of the Seebeck coefficient as the field increases. As a consequence, the overall variation of the Seebeck coefficient depends on the specific Fermi level of the individual bands and cyclotron frequency, and the Zeeman splitting affects the Seebeck coefficient particularly at high fields and low temperatures. Nevertheless, some discrepancies between the simplified model and experiments may be caused by the unavoidable temperature effect, which affects the scattering time and carrier density, as well as the Fermi energy in the practical experiment.

## Summary and outlook

In summary, the magneto-thermoelectric transport properties of a high-quality single-crystalline Bi_88_Sb_12_ topological insulator were systematically studied. A large magneto-Seebeck effect is observed due to the ultrahigh mobility and linear band dispersion, which leads to a gain in magneto-*zT* under magnetic fields as low as 1 T. Along with the reduced magneto-thermal conductivity, a high *zT* value of ~1.7 ± 0.2 is achieved at 180 K and 0.7 T. Rarely have materials exhibited such a high *zT*, particularly below 300 K; the magneto-thermoelectric correlation is important as an effective strategy to tune thermoelectric parameters synergistically. For practical applications, it is also essential to have a p-type thermoelectric material to complement the n-type crystal. Future studies should explore p-type Bi_1–*x*_Sb_*x*_ with hole doping, since the hole pockets of the Bi_1–*x*_Sb_*x*_ system are highly dispersive, promising a large magneto-thermoelectric response.

For future studies, we believe it is critical to control the mobility and Fermi level by precisely tuning the composition and purity of the elements, not only for the present Bi_1–*x*_Sb_*x*_ system, but also for other potential materials. For the Bi_1–*x*_Sb_*x*_ system, the Fermi level, which strongly affects the electron and hole compensation behaviour, is essential for decreasing the magneto-resistivity. Strategies can be considered, such as using high-quality raw Bi and Sb elements to avoid impurity doping, carefully deoxidizing and pre-zone melting the raw Bi to reduce the impurity density as much as possible and fine tuning the Fermi energy by parts-per-million-level doping. Most importantly, since the strong magneto-thermoelectric response benefits from ultrahigh mobility and a linear band dispersion, emergent topological materials showing such characteristics provide an intriguing platform for the advancement of thermoelectrics using the magneto-thermoelectric correlation strategy, which can even realize a non-saturating magneto-Seebeck effect when reaching the quantum limit.

## Methods

### Sample preparation

Bi_88_Sb_12_ single crystals were grown using a travelling molten zone melting technique within in-house equipment^38^. High-purity Bi (99.9999%) was used after pre-zone melting three times to reduce the impurity density. The material and quartz tubes were prepared to be as clean as possible in every step, and a low zone-melting rate of 1 mm h^–1^ was adopted.

### Sample characterization

The crystallinity and orientation of the as-grown single crystal were determined using electron backscatter diffraction. The as-grown single crystal cleaves easily in the (111) plane. Freshly cleaved samples were used for composition and homogeneity examination using scanning electron microscopy (Jeol JSM7800 F) with an energy dispersive X-ray (Quantax, Bruker) apparatus and electron backscatter diffraction system (CrystAlign, Bruker). Energy dispersive X-ray analysis demonstrated a chemical composition of *x* = 12 ± 1 in Bi_1–*x*_Sb_*x*_, which agreed well with the stoichiometric composition. After cutting samples into a bar shape using a wire saw, single crystals (~1.5 mm × 2 mm × 3.5 mm) were used to investigate the transport properties in the trigonal direction.

### Measurement of transport properties

Transport properties were measured by applying a current or temperature gradient along the trigonal direction, with a corresponding transverse magnetic field along the bisectrix direction. The resistivity (*ρ*) and Hall resistivity (*ρ*_H_) were measured using a physical properties measurement system (Quantum Design) with the electrical transport option. The Hall charge carrier concentration (*n*_H_) is determined by the slope of the magnetic field dependence of the Hall resistivity. The Hall mobility (*µ*_H_) values are calculated by *µ*_H_ = 1*/*(*e* × *ρ* × *n*_H_), where *e* is the elementary charge. The Seebeck coefficient and thermal conductivity were measured in the physical properties measurement system under high vacuum using a standard one-heater, two-thermometer steady-state method. The sample was fixed on a piece of heat sink with a strain gauge heater attached to the other end to apply a temperature gradient. Two sets of chromel–constantan thermocouples were mounted at two points along the temperature gradient to measure the temperature difference, and a chromel leg was used to measure the voltage difference. Electrical and thermal transport properties were measured on the same piece of sample with the same contacts, which helps eliminate the error in calculating *zT* (Supplementary Fig. 12). The measured raw data were field-symmetrized (for resistivity, Seebeck coefficient and thermal conductivity) and antisymmetrized (for Hall resistivity) to correct for contact misalignment. A series of samples were investigated, and the results of sample no. 1 are shown in the main text, whereas the results of samples no. 2 to no. 4 are shown in the Supplementary Information.

### Tight-binding modelling of band structure

To describe the band structure of Bi_1−*x*_Sb_*x*_ alloys, a tight-binding model is employed^38,39^. The initial parameters are derived from the unalloyed elemental metals bismuth and antimony, with their *s* and *p* orbitals included. Based on a modified virtual crystal approximation^38^, the band structure of Bi_1−*x*_Sb_*x*_ alloys depending on *x* can be well reproduced, as well as the evolution of band edges and the transition from semimetals to topological insulators.

### Modelling of magneto-Seebeck coefficient

In the present system, the electrons mainly come from the L points, which can be captured by the massive Dirac Hamiltonian, given by *H* = Δτ_3_ – *v*_*x*_*k*_*x*_*τ*_2_*σ*_1_ – *v*_*y*_*k*_*y*_*τ*_2_*σ*_2_ – *v*_*z*_*k*_*z*_*τ*_2_*σ*_3_ (ref. ^37^), wherein 2Δ is the bandgap, and *τ*_*i*_ and *σ*_*i*_ are Pauli matrices, and *k* and *v* with subscripts *x*/*y*/*z* are wave vector and the velocity of electrons along *x*/*y*/*z* direction, respectively. Without considering Zeeman splitting, the Seebeck coefficient at the weak field limit can be obtained by

$$S=\frac{{\uppi }^{2}{k}_{{\mathrm{B}}}^{2}T}{3e}\frac{{E}_{{\mathrm{F}}}}{({E}_{{\mathrm{F}}}^{2}-{\Delta }^{2})}\frac{2+{\left(\Delta /{E}_{{\mathrm{F}}}\right)}^{2}+3{\omega }_{{\mathrm{c}}}^{2}{{\rm{\tau }}}^{2}}{1+{\omega }_{{\mathrm{c}}}^{2}{{\rm{\tau }}}^{2}},$$

where ${E}_{{\mathrm{F}}}=\sqrt{{\Delta }^{2}+{\hslash }^{2}{{\widetilde{v}}_{{\mathrm{f}}}^{2}(6{\uppi }^{2}/{n}_{0})}^{2/3}}$ is the Fermi energy and ${\omega }_{{\mathrm{c}}}=\frac{{eB}{\widetilde{v}}_{{\mathrm{f}}}^{2}}{{E}_{{\mathrm{F}}}}$ is the cyclotron frequency, and ℏ and *n*_0_ are reduced Planck constant and intrinsic carrier concentration, respectively. Here, an effective Fermi velocity ${\widetilde{v}}_{{\mathrm{f}}}={({v}_{x}{v}_{y}{v}_{z})}^{1/3}$ is taken for simplicity. As an electron-dominated system, when the gap is negligible (Δ ≪ *E*_F_), the result is reduced to the case of a massless Dirac point^11^. Furthermore, there is a strongly anisotropic *g*-tensor in Bi_1−*x*_Sb_*x*_ alloys. The experimental *g*-value, as is well known and summarized by Shoenberg, shows a value of 244 with a cyclotron effective mass of 8.2 × 10^–3^ *m*_e_, for the light bisectrix band when the magnetic field is along the bisectrix direction^36^, in which *m*_e_ is the electron mass. This *g*-factor is even lower for the two heavy bisectrix bands, showing a half value of 122. In the case of *g* = 244, the Zeeman splitting energy *gμ*_B_*B* becomes ~10 meV at 0.7 T, in which *μ*_B_ is the Bohr magneton. Considering that the thermal broadening would be ~16 meV at 200 K, both of the individual bands are taken into account for the conduction, but with different Fermi energies. Similar is the case of *g* = 122. Here, to avoid an overestimation of the *g*-factor, *g* = 120 was adopted to calculate the Seebeck coefficient under a magnetic field along the bisectrix direction, by including an additional Zeeman term *H*_Z_ = *gμ*_B_*B* × *σ*. Here, the 2Δ gap is set to be 17 meV. The scattering time *τ* is set to be proportional to ${n}_{0}^{-1/3}$, and a value of 0.8 ps is set at 80 K for reference. The Fermi velocities are set to be *v*_*x*,*z*_ = 10^6^ m s^–1^ and *v*_*y*_ = 10^5^ m s^–1^.

## Online content

Any methods, additional references, Nature Portfolio reporting summaries, source data, extended data, supplementary information, acknowledgements, peer review information; details of author contributions and competing interests; and statements of data and code availability are available at 10.1038/s41563-024-02059-9.

## Supplementary information

Supplementary Information Supplementary Discussion and Figs. 1–12.

## Source data

Source Data Fig. 1 Source data for data plots.

Source Data Fig. 2 Source data for data plots.

Source Data Fig. 3 Source data for data plots.

Source Data Fig. 4 Source data for data plots.

## Data Availability

All the data supporting the plots within this paper and the findings of this study are available from the corresponding authors upon request. Source data are provided with this paper.
