# Supplementary material for: A magneto-thermoelectric with a high figure of merit in topological insulator Bi88Sb12
Source: Nat Mater. 2025 Jan 3;24(1):76–82. doi: 10.1038/s41563-024-02059-9 (PMC11698688; doi:10.1038/s41563-024-02059-9)
Supplement: Supplementary file 1 — Supplementary Discussion and Figs. 1–12. [file 41563_2024_2059_MOESM1_ESM.pdf]

nature materials

Supplementary information https://doi.org/10.1038/s41563-024-02059-9

A magneto-thermoelectric with a high figure of merit in topological insulator $Bi_{88}Sb_{12}$

In the format provided by the authors and unedited

Table of contents

Supplementary Text

1.1 Magneto-thermoelectric correlation……………………………………………..3

1.2 Thermoelectric properties of various samples……………………………..….3

1.3 Analysis of weak antilocalization contribution to magneto-*zT*…………….….4

1.3 Single crystal quality…………………………………………………………5

1.4 Composition homogeneity……………………………………………………6

1.5 Calculated magneto-Seebeck coefficient without Zeeman splitting…………6

1.6 Schematic illustration of the sample setup for transport properties measurements…………………………………..……………………………6

Supplementary Figures

Fig. S1 Magneto-thermoelectric properties correlation…………………………..7

Fig. S2 Thermoelectric properties of sample #2…………………………………8

Fig. S3 Thermoelectric properties of sample #3…………………………………8

Fig. S4 Thermoelectric properties of sample #4…………………………………9

Fig. S5 Thermoelectric performance of samples #1 and #2 compared to reported record values…………………………………………...…………………9

Fig. S6 Thermoelectric performance of samples #3 and #4 compared to reported record values…………………………………………………………………10

Fig. S7 Magneto-resistivity analysis at 180 K…………………………………..10

Fig. S8 Single crystal quality……………………………………………………11

Fig. S9 Microstructure image and composition homogeneity……………………11

Fig. S10 Elemental mapping of the as-grown crystal…………………………..12

Fig. S11 Calculated Seebeck coefficient without considering Zeeman splitting..12

Fig. S12 Schematic illustration of the sample setup for thermoelectric transport properties measurements……………………………………..………………13

References………………………………………………………………..………13

Magneto-thermoelectric correlation

The magnetic field affects the transport of charge carriers, particularly for high mobility systems that have strong magneto-responses, thus modulating the thermoelectric properties. Herein, we propose magneto-thermoelectric correlation as an effective strategy to tune three of the thermoelectric parameters for topological materials, particularly be able to enhance the magneto-Seebeck efficient and decrease the magneto-thermal conductivity simultaneously (**Fig. S1**(a)).

By semiclassical transport theory, the magneto-Seebeck coefficient is generally described by $\alpha = \alpha_\infty \mu^2 B^2/(1+\mu^2 B^2)+\alpha_0/(1+\mu^2 B^2)$, where α_0 is the Seebeck coefficients at zero magnetic fields, assuming resistivity ρ is magnetic field independent.[1] While note that, for topological materials with high μ, ρ is usually magnetic field dependent and the magneto-resistivity is large. For simplicity, **Fig. S1**(b) schematically shows the variation trends of the magneto-thermoelectric parameters with the assumption that $\alpha = \alpha_\infty \mu^2 B^2/(1+\mu^2 B^2)+\alpha_0/(1+\mu^2 B^2)$,[1] and $\rho = \rho_0(1+\mu^2 B^2)$,[2] using a mobility value of μ = 100000 cm^2/Vs, a ratio of α_∞/α_0 =2, and a lattice thermal conductivity of 2 W/mK. Clearly, there is an increased magneto-resistivity and magneto-Seebeck coefficient, and a decreased magneto-thermal conductivity owing to the reduced electronic part. The increase of Seebeck coefficient, decrease of electronic thermal conductivity, and the increase of resistivity competes on a delicate balance, which can lead to the increase of power factor and zT when high μ, large ratio of α_∞/α_0, and low lattice thermal conductivity are satisfied. When the increase of resistivity surpasses the positive contribution from enhanced magneto-Seebeck coefficient, the zT decreases at elevated magnetic fields. **Fig. S1**(c)–(f) further shows the general trends of magnetic field dependence of the Seebeck coefficient, resistivity, thermal conductivity, power factor and zT for different mobilities, respectively. As indicated by the blue lines, thermoelectric parameters barely changed under a magnetic field with low mobility. For intermediate-high mobility, the Seebeck coefficient and resistivity were enhanced along with a decrease in electronic thermal conductivity, as indicated by the green lines. For ultrahigh mobility (100 times higher), the requirement of an external magnetic field to obtain a high Seebeck coefficient can be reduced to a much lower value, as illustrated by the red line in **Fig. S1**(c). Therefore, high mobility causes a rapid increase in the power factor and zT value under a low magnetic field (**Fig. S1**(e) and (f)).

Thermoelectric properties of various samples

In order to explore the origin of the high thermoelectric performance, and to demonstrate the role of mobility for high thermoelectric performance of the $Bi_{88}Sb_{12}$ single crystals, as well as to investigate the reproductivity, four samples were measured.

The thermoelectric transport properties of sample #1 are shown in the main text, and the thermoelectric transport properties of samples #2 - #4 are shown in **Fig. S2-S4**, respectively. Comparing the thermoelectric properties of all the samples, it is found that the magneto-Seebeck coefficient values are almost identical, while the magneto-resistivity can have a large difference, which therefore leads to a large difference in magneto-*zT*. We attribute the different magneto-resistivity to the sensitive perturbation of the charge carriers transport by various scattering process. As such, we expect even better thermoelectric performance by further well controlling the single crystal quality.

We then show the performance improvements of the present work compared to the previously reported record value by Wolfe and Smith.[3] As shown in **Fig. S5** and **Fig. S6**, all samples have achieved great improvements in z compared to the previous work, far beyond the error bar. Furthermore, we have also presented the optimal field to obtain the z_{max}, from which we find that the optimization field decrease rapidly with decreasing temperature and varies from sample to sample.

Analysis of weak antilocalization contribution to magneto-*zT*

We further compared the magneto-resistivity that essentially affect the magneto-*zT* by taking sample #1 (zT_{max}~1.9) and sample #3 (zT_{max}~1.3) as examples. It is found that samples #1 and #3 present a different magneto-resistivity behavior. As shown in **Fig. S7**(a), S#1 shows a nearly quadratic increase resistivity with magnetic field, but with a tiny dip at very low magnetic field. We fitted the magneto-resistivity of sample #1 using the formula $\rho = \rho_0(1+ \mu^2B^2)$, the fitted line is shown by the grey dash line in **Fig. S7**(a). A fitted mobility of μ = 10000 cm^2/Vs is adopted. It is found that the experimental magneto-resistivity diverges from the quadratic relation $\rho = \rho_0(1+ \mu^2B^2)$ to some extent. In contrast, sample #3 barely present a quadratic behavior but a mustache-shaped magneto-resistivity with a more obvious dip at the low magnetic field. The dip of the magneto-resistivity at the low magnetic field is usually recognized as a signature of the existence of two types of charge carriers with different mobilities, or weak antilocalization effect.

Here we use the Hikami-Larkin-Nagaoka (HLN) model to investigate if there is weak antilocalization effect that differs and magneto-resistivity and thus the magneto-*zT*. According to the HLN model:[4,5]

$$\Delta\sigma/\sigma(0) = \frac{\sigma(B) - \sigma(0)}{\sigma(0)} = C\left(\ln\frac{B_\phi}{B} - \psi(\frac{1}{2} + \frac{B_\phi}{B})\right)$$

In which σ is conductivity, C is a constant, ψ is the digamma function, and B_ϕ is the phase coherence characteristic field. Then it is possible to evaluable the phase coherence length L_ϕ by:

$$B_\phi = \frac{\hbar}{4eL_\phi^2}$$

As shown in **Fig. S7**(b), the experimental mustache-shaped part of the change of conductivity can be well fitted by the HLN model. It is found that the coherence length L_ϕ of both samples is almost identical, showing a value about 100 nm. Moreover, we compared the L_ϕ to the mean free path of the electrons L_e, since the quantum interference is only effective when the $L_\phi < L_e$. Based on the Drude model,[6] the mean free path can be resolved from the conductivity by knowing the Fermi wave vector k_F:

$$\sigma(0) = \frac{e^2}{3\pi^2\hbar} k_F^2 L_e$$

In which $k_F = \sqrt[3]{3\pi^2 \mathrm{n}}$ for a bulk crystal, and n is the electron concentration which is taken as the Hall electron concentration in this work. Here as the comparison of different samples are of the same crystal, the anisotropy of the Fermi surface that is not taken into account would not significantly affect the conclusion. The obtained L_e is as large as 1200-1700 nm at 180 K, which is much larger than the L_ϕ, demonstrating the effectiveness of the weak antilocalization effect in all the samples. Quantitively, the correction to the magneto-resistivity due to weak-antilocalization is proportional to (-$1/L_e + 1/L_\phi$), where L_ϕ and L_e are the quantum coherence length and mean free path of the electrons, respectively. Evidently, a larger L_e (or higher mobility) produces a larger enhancement in magneto-zT. To understand how much gain can be achieved for magneto-zT enhancement from the weak antilocalization effect, we go through the basic integral equation below,[7]

$$\frac{\Delta\sigma}{\sigma} = -\frac{\lambda^2 v}{D^{3/2}t^{3/2}} \int_\tau^{\tau_\varphi} (\frac{3}{2} e^{-\frac{t}{\tau_{so}}} - \frac{1}{2}) dt$$

$$= \frac{\lambda^2 v}{D^{\frac{3}{2}}} [\left(\frac{1}{\sqrt{\tau}} - \frac{1}{\sqrt{\tau_\varphi}}\right) + 3(\frac{1}{\sqrt{\tau_\varphi}} e^{-\frac{\tau_\varphi}{\tau_{so}}} - \frac{1}{\sqrt{\tau}} e^{-\frac{\tau}{\tau_{so}}})]$$

in which $\lambda = \frac{a}{\pi}, v = \frac{\pi\hbar}{am^*}$, $D = lv = v^2\tau$, a is the lattice parameter, m^* is the effective mass, respectively. By plugging the numbers τ = 10^{-12} s, τ_{SO} = 10^{-13} s, and τ_φ = 10^{-11} s, we found the change in $\frac{\Delta\sigma}{\sigma}$ is very small (in the order of 10^{-12}). In this case, we clarify that quantitively weak antilocalization has a small contribution to the conductivity change, so as the zT change.

Single crystal quality

The crystals of this work are of high quality. As shown in **Fig. S8**, the crystal is

millimeters large with a shining cleavage plane. The cleavage plane is (111) plane. IPF Z (inverse pole Figure Z) images further demonstrates the high quality of the single crystallinity, as shown in **Fig. S8**. Here the [001] direction in EBSD is the *c*-axis of $Bi_{88}Sb_{12}$ in hexagonal structure, which is identical to the [111] direction in rhombohedral structure. The single crystal quality becomes better in the middle part (left part in **Fig. S8**) than the beginning part (right part in **Fig. S8**) along the as-grown crystal.

Composition homogeneity

The composition and homogeneity of the crystal were examined by energy-dispersive X-ray spectroscopy (EDX) and EBSD. **Fig. S9** show the backscattered electrons (BSE) images and EDX results, which confirm the uniform distribution of the elements and indicate the homogeneous elemental composition ratio of Bi:Sb close to 88:12, or 89:11, which agrees well with the stoichiometric ratio.

The elemental mapping along with elemental ratio analysis of the single crystals, and the electron images recorded with the forescattered electron detector of the EBSD system are shown in **Fig. S10**. The results demonstrate the Bi and Sb elements are almost homogenous distributed in the single crystal, with a ratio close to the stochiometric ratio. Slight differences are observed because Bi and Sb form solid solution with a large segregation coefficient.

Calculated magneto-Seebeck coefficient without Zeeman splitting

Using a massive Dirac Hamiltonian, the calculated results without considering Zeeman splitting are shown in **Fig. S11**. It is found that the Seebeck coefficient is enhanced by the magnetic field owing to the increase of cyclotron frequency. Meanwhile, the raised Fermi level, together with the reduced relaxation time, contributes to the increase of saturation fields at higher temperatures.

Schematic illustration of the sample setup for transport properties measurements

The electrical and thermal transport properties are measured on the same piece of sample with the same contacts, which helps eliminate the error in calculating zT (**Fig. S13**). Taking the measurements along the trigonal direction as an example, the resistivity and Hall resistivity are measured simultaneously via V_1 and V_2 by applying a current along the trigonal direction, respectively. The Seebeck and thermal conductivity are measured simultaneously by detecting V_1, T_H, and T_C, as well as the heater power, by applying a temperature gradient along the trigonal direction.

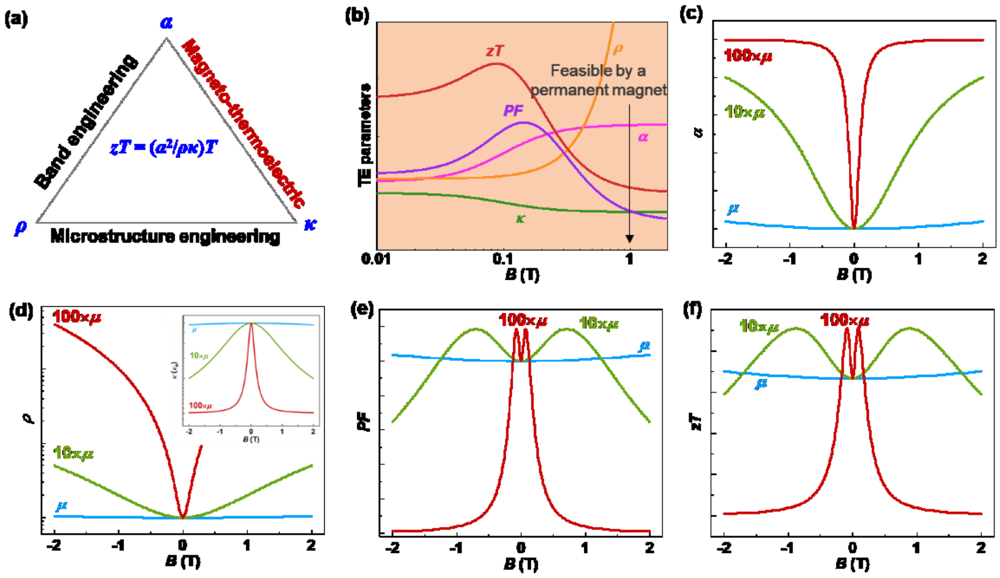

Fig. S1 Magneto-thermoelectric properties correlation. (a) Schematic of magneto-thermoelectric correlation to simultaneously modulate the thermoelectric parameters including Seebeck coefficient α and thermal conductivity κ. (b) Modelling of magneto-thermoelectric correlation to simultaneously modulate the thermoelectric parameters including resistivity ρ, Seebeck coefficient α, thermal conductivity κ, power factor PF, and zT. The values of ρ, α, κ, PF, and zT are presented for a variation trend without absolute values. (c) Seebeck coefficient. (d) Resistivity, inset shows thermal conductivity. (e) Power factor. (f) zT. Three different mobilities increasing from μ to 10 μ to 100 μ (μ = 1000 cm^2/Vs) are illustrated. The ratio of $\alpha_\infty/\alpha_0 = 2$. A lattice thermal conductivity of 2 W/mK is adopted. The higher the mobility, the smaller the magnetic field required for a large zT.

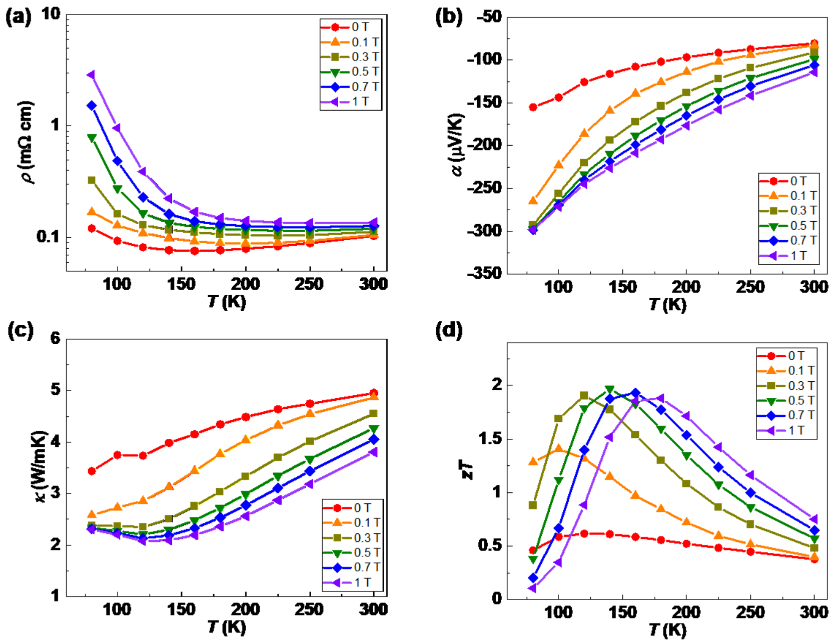

Fig. S2 Thermoelectric properties of sample #2. Temperature dependence of the thermoelectric transport properties. (a) resistivity, (b) Seebeck coefficient, (c) total thermal conductivity, and (d) zT values of sample #2 under 0-1 T.

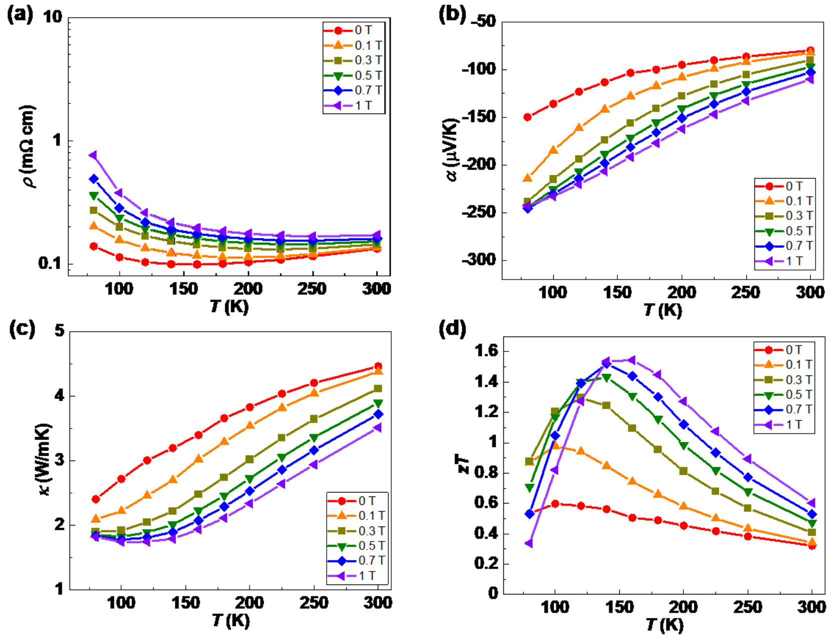

Fig. S3 Thermoelectric properties of sample #3. Temperature dependence of the thermoelectric transport properties. (a) resistivity, (b) Seebeck coefficient, (c) total thermal conductivity, and (d) zT values of sample #3 under 0-1 T.

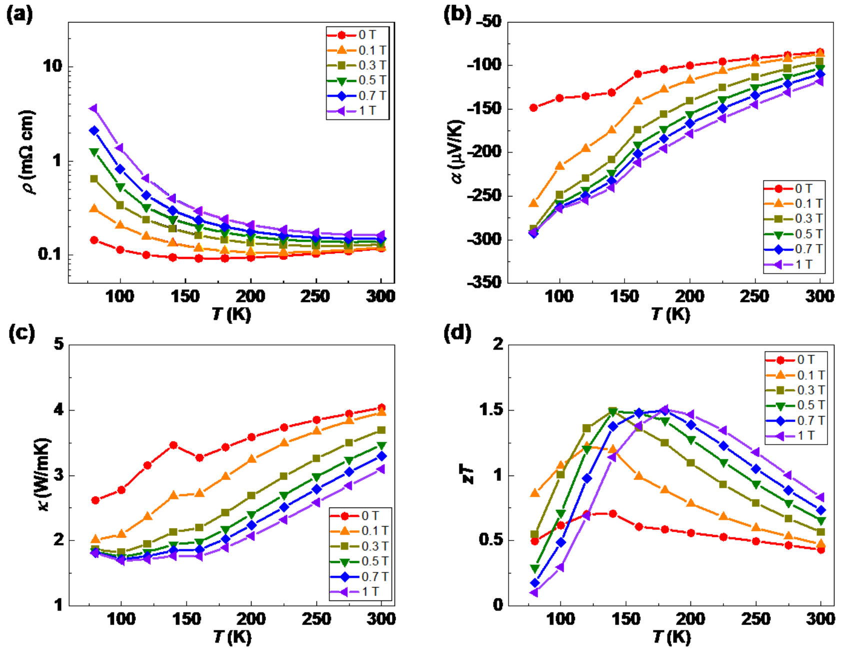

Fig. S4 Thermoelectric properties of sample #4. Temperature dependence of the thermoelectric transport properties. (a) resistivity, (b) Seebeck coefficient, (c) total thermal conductivity, and (d) zT values of sample #4 under 0-1 T.

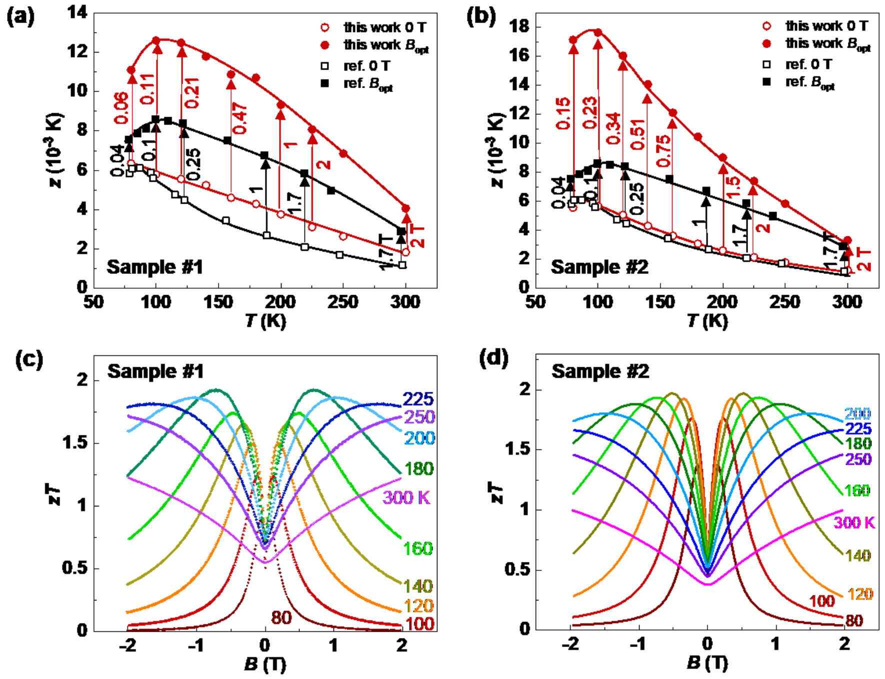

Fig. S5 Thermoelectric performance of samples #1 and #2 compared to reported record values. Temperature dependence of the thermoelectric z value for (a) sample #1, (b) sample #2. Magnetic field dependence of zT for (c) sample #1, (d) sample #2.

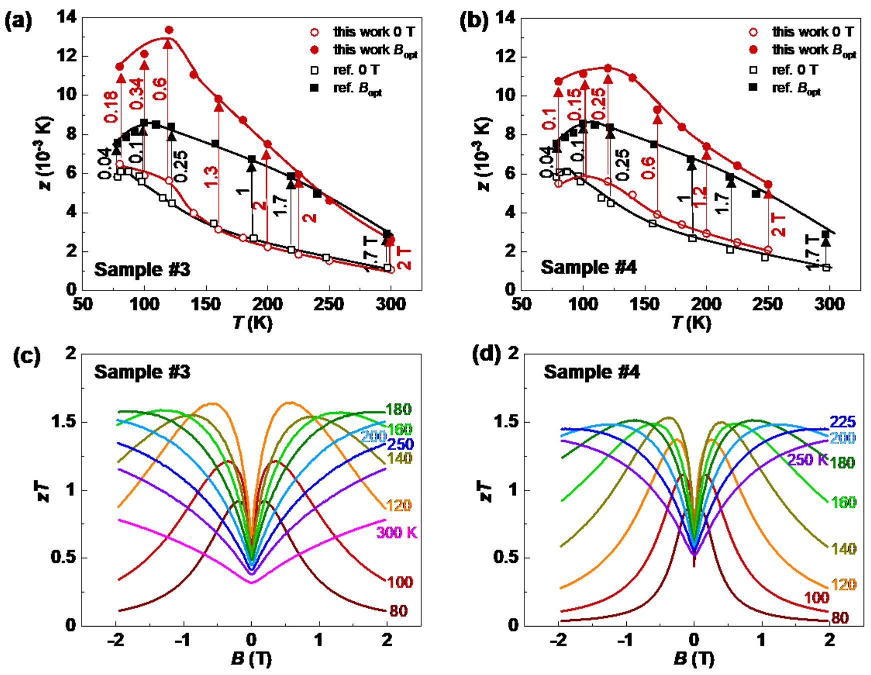

Fig. S6 Thermoelectric performance of samples #3 and #4 compared to reported record values. Temperature dependence of the thermoelectric z value for (a) sample #3, (b) sample #4. Magnetic field dependence of zT for (c) sample #3, (d) sample #4.

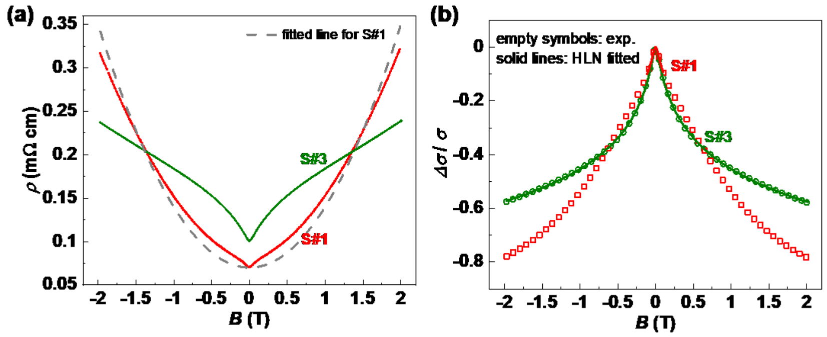

Fig. S7 Magneto-resistivity analysis at 180 K. (a) Magneto-resistivity and (b) fitting of the change of conductivity as a function of magnetic field by the Hikami-Larkin-Nagaoka (HLN) model for sample #1 and #3. The grey dash line in (a) is fitted for sample #1 using $\rho = \rho_0(1+\mu^2 B^2)$.

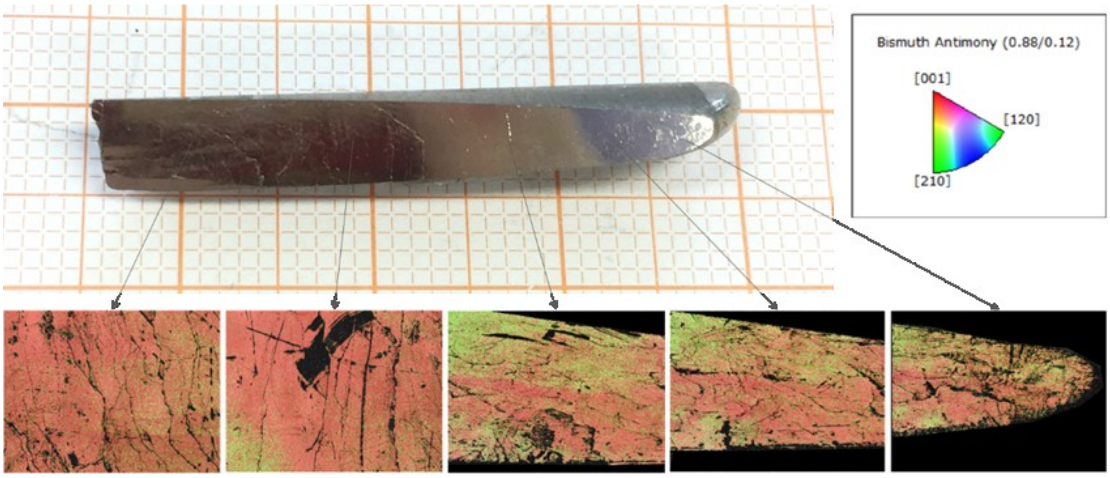

Fig. S8 Single crystal quality. Optical image (top left), and color coded orientation of the unit cell of the $Bi_{1-x}Sb_x$ phase perpendicular to the cleaving plane at different areas along the as-grown crystal (inverse pole figure Z, IPF Z) (bottom images, with color code in the hexagonal setting shown on top right). The crystal was millimeters in size with a shining cleavage plane of high single crystal quality.

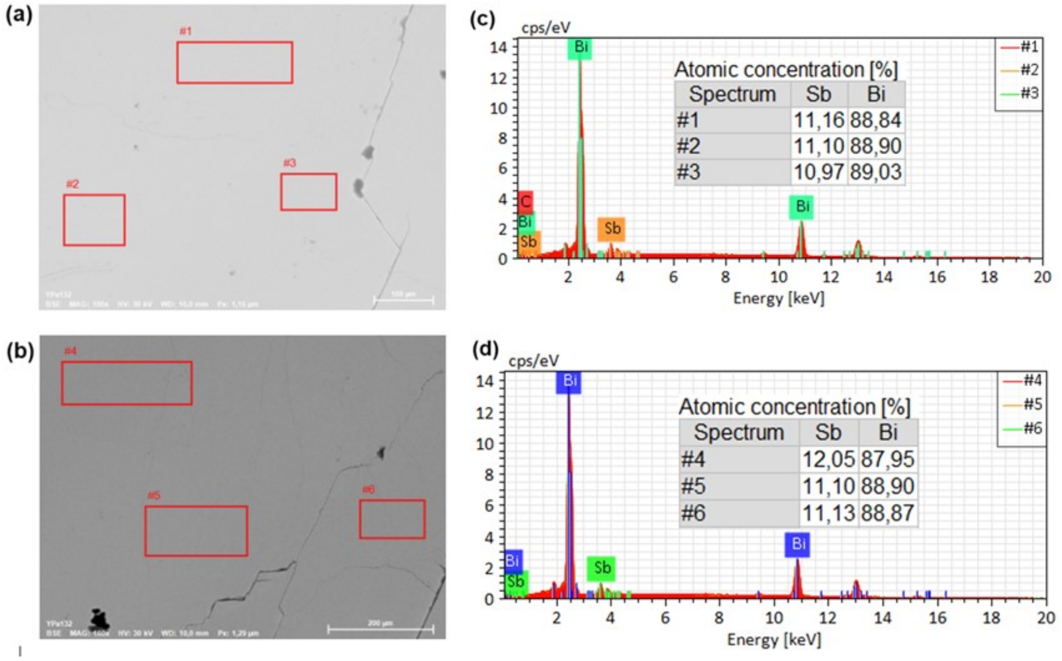

Fig. S9 Microstructure image and composition homogeneity. (a), (b) Backscattered electron (BSE) image and (c), (d) energy-dispersive X-ray spectroscopy (EDX) analysis (acc. voltage 30 kV) at various areas on the $Bi_{88}Sb_{12}$ single crystal.

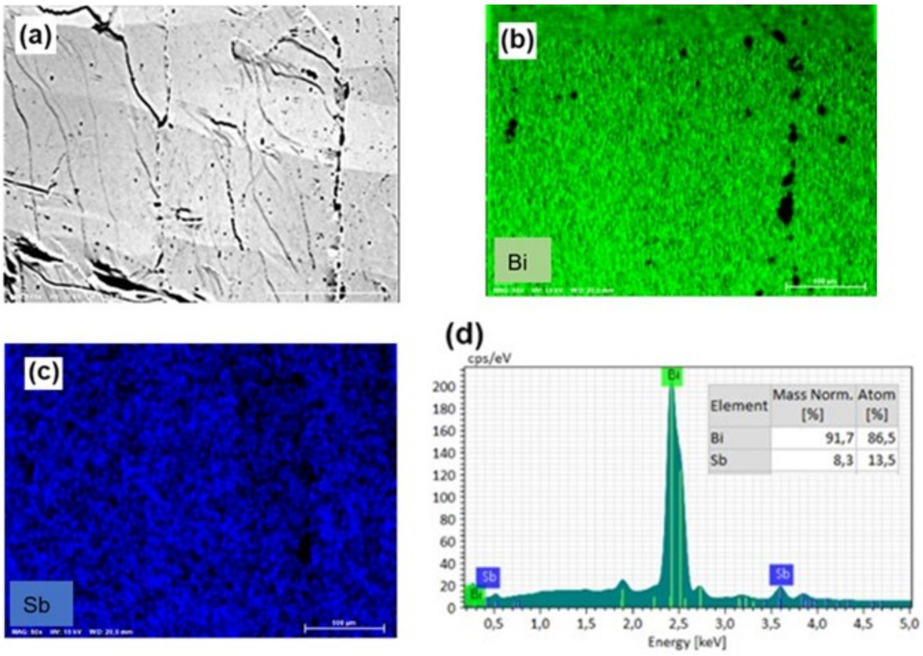

Fig. S10 Elemental mapping of the as-grown crystal. (a) Fore-scattered electron image (70° tilt corrected) on the cleaved surface with (111) orientation of the crystal. Material contrast and element mapping images show the homogeneity of the single crystalline sample. Black lines and particles originate from terraces steps and cleaving residues, respectively. X-ray intensity distributions of (b) Bi $M\alpha$ (2.4 keV) and (c) Sb $L\alpha$ (3.6 keV). (d) Element concentration calculated from X-ray intensities (15 kV, ZAF matrix correction model) of the energy dispersive spectra.

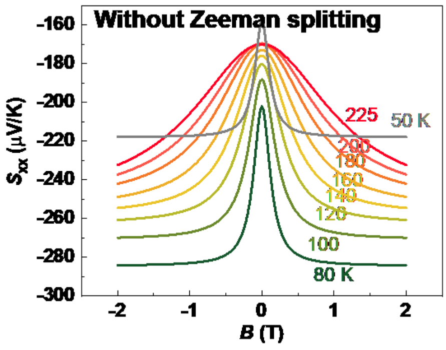

Fig. S11 Calculated Seebeck coefficient without considering Zeeman splitting. Compared to the results with Zeeman splitting, the Seebeck coefficient here exhibits a significant enhancement and saturates at low temperatures as the field increases.

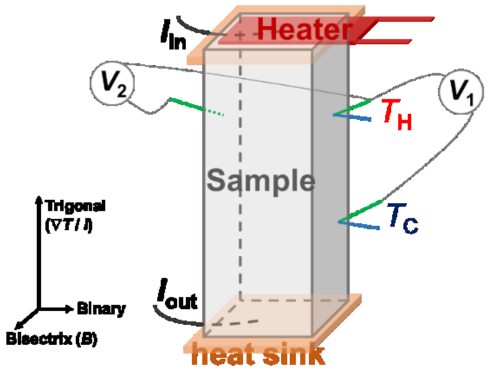

Fig. S12 Schematic illustration of the sample setup for thermoelectric transport properties measurements. The sample was fixed on a piece of heat sink with a strain gauge heater attached to the other end to apply a temperature gradient. Two sets of chromel-constantan thermocouples were mounted at two points along the temperature gradient to measure the temperature difference (T_H and T_C), and a chromel leg was used to measure the voltage difference (V_1). A transverse voltage difference V_2 is measured for Hall voltage.

References

1. T. Liang, J. Lin, Q. Gibson, T. Gao, M. Hirschberger, M. Liu, R. J. Cava, N. P. Ong, Anomalous Nernst effect in the Dirac semimetal Cd3As2. *Phy. Rev. Lett.* **118**, 136601 (2017).
2. H. Weiss, H. Welker, Zur transversalen magnetischen Widerstandsänderung von InSb. *Z. Physik* **138**, 322–329 (1954).
3. R. Wolfe, G. E. Smith, Effects of a magnetic field on the thermoelectric properties of a bismuth-antimony alloy. *Appl. Phys. Lett.* **1**, 5-7(1962).
4. H. Hayasaka, Weak antilocalization in spin-orbit coupled lattice systems: Effect of nonadiabatic transitions and estimation of spin relaxation length. *Phys. Rev. B* **104**, 205404 (2021).
5. D. M. Vu, W. Shon, J.-S. Rhyee, M. Sasaki, A. Ohnishi, K.-S. Kim, H.-J. Kim, Weak antilocalization and two-carrier electrical transport in $Bi_{1-x}Sb_x$ single crystals (0%≤ x≤ 17.0%). *Phys. Rev. B* **100**, 125162 (2019).
6. N. W. Ashcroft, N. D. Mermin, *Solid State Physics* (Holt, Rinehart and Winston; New York, 1976).
7. A. A. Abrikosov, Fundamentals of the theory of metals. Dover edition, Garden City, New York (2017).
